# Safety, Feasibility, and Preliminary Efficacy of Allogeneic MSCs to Treat Advanced Femoral Head Osteonecrosis (ALOFEM): A Pilot Study in Young Onco‐Hematological Patients

**DOI:** 10.1155/sci/1986839

**Published:** 2026-01-05

**Authors:** Enrique Gómez-Barrena, Norma G. Padilla-Eguiluz, Juan Cabello-Blanco, José Juan Pozo-Kreilinger, Yasmina Mozo-del-Castillo, María E. Martínez-Muñoz, Trinidad Martín-Donaire, Rocío Zafra, Rafael F. Duarte, Ana Velasco-Iglesias, Cristina Avendaño-Solà, Concepción Payares-Herrera

**Affiliations:** ^1^ Department of Orthopedic Surgery, La Paz University Hospital-IdiPAZ, Madrid, Spain, idipaz.es; ^2^ Department of Surgery, School of Medicine, Autonomous University of Madrid-IdiPAZ, Madrid, Spain; ^3^ Department of Pathology, La Paz University Hospital, Madrid, Spain, hulp.es; ^4^ Department of Pediatric of Onco-Hematology, La Paz University Hospital, Madrid, Spain, hulp.es; ^5^ Department of Hematology and GMP Cell Production Unit, Puerta de Hierro Majadahonda University Hospital, Madrid, Spain, madrid.org; ^6^ Department of Clinical Pharmacology, Puerta de Hierro-Majadahonda University Hospital, Madrid, Spain, madrid.org

**Keywords:** allogenic bone marrow derived MSC, expanded MSCs, osteonecrosis of the femoral head (ONFH), pilot phase 1 clinical trial

## Abstract

**Background:**

Severe osteonecrosis of the femoral head (ONFH) secondary to corticosteroid therapy in symptomatic, hematological young patients currently has no therapeutic alternative, and early total hip replacement (THR) is a high‐risk intervention in those patients.

**Objectives:**

To evaluate feasibility, safety, and early efficacy of allogeneic expanded mesenchymal stromal cells (MSCs) in a pilot clinical trial.

**Methods:**

Pilot phase 1 open, noncontrolled, nonrandomized clinical trial evaluating the bone regeneration capacity in seven hips from four patients (young females 11–19 year old) with symptomatic, severe bilateral femoral head osteonecrosis (secondary to corticosteroid therapy), 1 year after being surgically treated with 140 × 10^6^ allogenic MSC plus forage.

**Results:**

The proposed therapy proved feasibility, safety at 1 and 4 years (no related serious adverse events [SAEs]), and early efficacy (nonsignificant) in the case analysis (5/7 hips avoiding THR at 4 years).

**Conclusions:**

The implantation of expanded allogeneic MSC in young patients to prevent conversion to a THR or collapse of the femoral head due to severe osteonecrosis is feasible without safety concerns in the longer‐term follow‐up (FU) upto 4 years.

**Trial Registration:** EudraCT number: 2018‐000886‐35

## 1. Introduction

Orthopedic surgeons are looking for innovative therapies when bone necrosis threatens the bone structure and function, to launch bone regeneration through tissue engineering [1] and tissue repair based on cell therapy [2]. One developed approach consists of harvesting bone marrow (BM) from the patient, isolating mesenchymal stromal cells (MSCs) by their plastic adherence, expanding the adherent cells to a sufficient number, stimulate their differentiation towards osteogenesis and then seeding them onto a suitable synthetic scaffold prior to re‐implantation [3, 4]. Implanting cells to augment the host cells after differentiation in the damaged bone has been proposed to procure osteogenesis [5].

Osteonecrosis of the femoral head (ONFH), whether idiopathic or due to steroids, alcohol abuse, or other causes, is a difficult‐to‐treat problem. Idiopathic and alcohol‐related ONFH occurs mostly in middle‐aged males, but it may also occur in children, young and older adults, particularly in the context of high dose corticosteroid treatment (as part of the protocols to treat hematologic malignancies or other major diseases) [6, 7]. The ONFH secondary to high‐dose steroid treatment may progress to femoral head collapse and early hip destruction, with rapid osteoarthrosis even at very early age. This may then lead to hip pain resulting in long and fastidious treatments, substantial socioeconomic costs, and even permanent disability. The only approach at this final stage is total hip replacement (THR).

ONFH healing in early stages is infrequent, due to bone necrosis and low number of viable osteogenic cells in the damaged bone. Hernigou and Beaujean [8] found a decrease in the number of MSCs in the upper end of the femur in patients with corticosteroid‐induced osteonecrosis. Therefore, the avoidance of femoral head collapse in ONFH through newly formed bone support remains a challenge. The implantation of a large number of BM derived human MSCs, isolated, cultured, expanded, and differentiated into osteoblastic lineage under GMP (good manufacturing practices) conditions, appears to be a reasonable approach that may contribute to enhance the osteogenic activity of the host bone [8]. The implantation of high dose (20 × 10^6^ MSC/mL) autologous expanded BM derived MSC has been tested by our research group in pseudarthrosis or nonunions of long bone fractures and in femoral head osteonecrosis.

The ORTHO‐2 clinical trial using GMP‐standardized manufacturing techniques demonstrated that implantation of 140 × 10^6^ autologous MSC plus forage failed in only 4 out of 22 cases, requiring a THR before 12 months after surgery. Long‐term follow‐up (FU; upto 5 years) showed no progression in 16 of 21 cases [9]. Additionally, the osteonecrosis volume (ONV) significantly decreased from 18.7% at baseline to 11.6% at 3 months, and to 3.7% after 1 year. However, in the cases that progressed to the next stage or received THR, the ONV decreased but not enough to achieve a lesion under a mean 10% (SD 6%) at 3 months FU [10]. These results suggest the therapeutic potential of high doses of autologous MSCs for treating ONFH. However, patients with hematological diseases requiring hematopoietic stem cell transplantation (HSCT) may lack sufficient BM progenitors, being donor‐derived MSCs necessary for effective bone regeneration through localized injections of an adequate number of expanded MSCs with preserved functional capacity.

In the last decade, intravenous (i.v.) infusions of donor (allogeneic) MSC have been used in patients with various conditions in clinical studies, most of them using expanded cells grown with fetal calf serum (FCS) and cryopreserved with dimethyl sulfoxide (DMSO), both with and without control groups, including randomized study designs. A meta‐analysis of results from properly reported studies performed using allogeneic MSC from HLA‐matched or mismatched donors in patients with various conditions has been performed by Lalu et al. [11]. In this meta‐analysis, transient fever without major clinical relevance was a frequent observation following the i.v. infusion of culture expanded MSC, in most cases grown with FCS and given after cryopreservation and thawing. No significant association was detected between MSC use and development of any other acute infusional toxicity, organ system complications, infection, death, or malignancies.

The i.v. infusions of expanded MSC from the same HSCT donor or a different one, most often HLA mismatched, is currently a common practice to treat patients developing graft‐versus‐host disease (GVHD) after HSCT, with favorable effects in a high proportion of patients, seemingly related to the immunoregulatory and cytokine mediated anti‐inflammatory effects of the MSC. This mechanism of action may raise concerns about the possibility of favoring the development of infections or relapse of the hematological malignancy treated by means of allogeneic HSCT [12].

The so far accumulated clinical experience has not denoted, though, that MSC infusions increase neither the incidence of severity of infections, which is an inherent risk in such immunocompromised patients. Nor is there clear evidence of increased incidence of relapses or development of other de novo malignancies in the pooled data of the overall experience of treating GVHD in those patients with cultured expanded MSC. Specifically, the overall experience has not yielded so far any evidence of tumor development from the infused expanded MSC. All these possible events are in fact a matter of close scrutiny in patients of this nature being treated with i.v. infusions of MSC. On the other hand, MSC therapy has not been associated with the development of immune allo‐reactions in this context, neither in graft‐versus‐host (GVH) nor host‐versus‐graft (HVG) directions. MSCs are actually known to have low immunogenicity due to their poor expression of MHC and T‐cell co‐stimulatory molecules, more so in the context of immunosuppressed recipients.

Moreover, it is worth noting that the use of allogeneic MSCs, instead of autologous, may have several practical advantages. Among those, it is accepted that it would provide easy availability when needed, and in the case of patients with comorbidities, the possibility of using a product richer in MSC than what can be obtained from these. Thus, the type of patients elected for the study is most suitable to evaluate the osteogenic potential of allogeneic expanded MSC.

A challenging situation is the selection of pediatric subpopulation (under 18) to be included in the study. Like in adults, ONFH also occurs in children, adolescents and young adults as a known complication of high dose corticosteroid treatments, in the context of the treatment of hematologic malignancies or other major diseases [7]. Furthermore, considering the limited options to treat ONFH and the potential disastrous consequences of this disorder (e.g., permanent disability and requirement of THR in an early age), young patients do not have a current standard therapy for this condition. Bearing in mind the proposed dose in this study and mode of administration (single dose, locally administered in the femur head) and the available pre‐clinical and clinical data, no systemic effects, including potential deleterious effects on growth and development, were expected for the investigational medicinal product (IMP). Therefore, the potential risks for children and adolescents were not expected to substantially differ from those expected for adult patients, which would allow to plan a pilot clinical trial. Furthermore, there are no available clinical data from female adolescents suffering from this condition, and the focus of a clinical trial could be this subpopulation.

We hypothesized that a single injection of a high dose of allogeneic BM‐derived, in vitro expanded MSCs may safely contribute to the improvement of ONFH and that the proposed method is feasible, even in young female patients, where no current data are available.

## 2. Materials and Methods

### 2.1. Study Design and Participants

A phase I/IIa open, nonrandomized, prospective, interventional pilot clinical trial named ALOFEM (Eudra No. 2018‐000886‐35) was an independent, frontier initiative to develop a treatment for complex cases of ONFH in patients with a history of HSCT to treat a previous hematological disease, and was conducted in Madrid, Spain, from 2020 to 2022. The study was approved by the Puerta de Hierro University Hospital Research Ethics Committee (No. 39.18) and authorized by the Spanish Agency of Medicines and Medical Devices. The Spanish Competent Agency also authorized the compassionate use for cases 103 and 104 (codes: SLC448901019026 and SLC863701266511, respectively).

The primary objective was safety and feasibility of BM‐derived allogeneic human MSC used in ONFH with placement at the osteonecrosis site. The secondary objective was to confirm bone regeneration in the femoral head, maintaining head sphericity and/or avoiding progression towards femoral head collapse and THR, without increasing the complication rate, in hips with symptomatic ONFH.

#### 2.1.1. Selection Criteria for Patients

The inclusion criteria were age 8–55, both sexes, with symptomatic ONFH (ARCO stages 0, I, IIA, IIB, IIC, IIIA, IIIB, IIIC or Steinberg stages 0, I, II, III), in patients having received HSCT due to hematologic disease (in remission), with or without immunosuppression. Patients had signed informed consent (by themselves or legal representatives). Table S1 lists the complete selection criteria for candidate patients.

#### 2.1.2. Selection Criteria for Donors

Donors were selected from the donor assessment performed for the patient’s previous HSCT. The inclusion criteria were age 18–55, both sexes; able to provide and signed informed consent for BM donation, preservation, and clinical use of the cell therapy; with medical coverage under the Spanish National Health System; ability to understand and accept the study constraints; and nonidentical HLA. The exclusion criteria were active infection of any location and etiology; pregnancy or breastfeeding women; diabetes; blood disease of any origin; any chronic debilitating disease; use of immunosuppressive drugs; active tumor (previous or concomitant); known positivity for HBsAg, anti‐HBc IgM, anti HCV, anti‐HIV‐1 or 2, or syphilis, or not fulfilling the national requirements for donors.

#### 2.1.3. Participant Cases

Seven hips were treated from four symptomatic female patients in the Onco‐Hematology and Orthopaedic Department of La Paz University Hospital, with a history of HSCT with bilateral ONFH due to corticosteroid treatment associated with their hematological malignancy (Table 1). Four hips were left‐sided and three right‐sided (codification of the case number is presented, ending in L for left and R for right). Four hips from two patients (cases 101 and 102) were selected from seven screened cases under the ALOFEM clinical trial. The main exclusion criteria in the nonselected cases were relapse of their hematological disease (*n* = 4) and being under corticoid therapy without possibility to stop it (*n* = 1). Three hips from two patients (cases 103 and 104) were compassionately treated following the same procedures and have been included in this report. See Figure S1.

**Table 1:** Demographics and general characteristics of the ALOFEM study cases.

Variable	Case 101	Case 102	Case 103	Case 104
Demographics				
Age	19	13	14	20
Sex	Female	Female	Female	Female
Body mass index	21	23	18	20
Alcohol consumption	No	No	No	No
Smoking habit	No	No	No	No
Onco‐hematological disease history				
Hematological disease history (in remission)	Myeloblastic leukemia	Severe bone marrow aplasia	Lymphoblastic leukemia pre‐B associated to hyper‐eosinophilia	Lymphoblastic leukemia
Hematopoietic progenitor cell transplantation history (donor)	HLA identical (sibling)	HLA identical (sibling)	HLA haplo‐identical(parent)	HLA identical (sibling)
Osteonecrosis of the femoral head (ONFH) characteristics				
ON possible cause	Corticosteroid treatment	Corticosteroid treatment	Corticosteroid treatment	Corticosteroid treatment
Bilateral	Yes	Yes	Yes	Yes
ARCO stage (left/right)	IIIA/IIIB	IIIB/IIIA	IIIA/IIA	IIIA/IIB
Included hip laterality	Both	Both	Right	Both

Of the seven hips treated and analyzed, case 102R, exhibited a more severe ONFH stage according to Steinberg classification (stage IV) but met the selection criterion according to ARCO staging; this protocol deviation was reviewed and approved by the study sponsor. Follow‐up (FU) of compassionate treated case 103R was lost at 18 months because of relapse of her lymphoblastic leukemia and subsequent exitus, that was considered unrelated to the investigational treatment. Cases 104L and 104R were clinically and radiologically followed in a different clinical center; therefore, MRI tests were unavailable after 3 months. All treated patients received medication due to complications of their hematological disease during their participation in this study. Corticotherapy was required in the hematological FU of cases 101 and 103, at 2 and 6 months, respectively.

### 2.2. Procedures

#### 2.2.1. IMP

BM harvesting from donors was performed in compliance with the Cells and Tissues Directives following the methods validated in the REBORNE EU‐funded project (regenerating bone defects using new biomedical engineering approaches, FP7 HEALTH‐2009‐1.4‐2, Grant Agreement 241879) and published by our group [5, 13]. Specifically, donors needed to be negative in serology for anti‐HIV 1–2 Ab, anti‐HCV Ab, HBs Ag, anti‐HBc syphilis, and negative (not detected by PCR) in HIV NAT, HCV NAT, or HBV NAT. The entire manufacturing process was performed at the GMP Cell Production Unit of the Department of Hematology of Puerta de Hierro Majadahonda University Hospital. For the production process, approximately 30 mL of BM were aspirated from the donor’s iliac crest under general or regional anesthesia following a standard procedure. BM was transported to the GMP facility in less than 2 h and with controlled temperature in all cases. The starting material was seeded directly in alpha‐MEM culture medium supplemented with human platelet lysate, and MSC were obtained and expanded in a humidified atmosphere with 5% CO_2_ at 37°C using a two‐step procedure of P0 for 14 days and P1 for 7 days. Thus, 21 days were needed to produce one batch of MSCs for therapeutic use. The identity of BM MSCs was confirmed in the GMP facility in the validation phase by evaluating MSC markers, following the minimum criteria of the International Society for Cellular Therapy guidelines. MSCs were characterized by flow cytometry positive for surface antigens CD73, CD90, and CD105 and negative for CD45, CD34, and HLA class II. Defined validations and quality controls for sterility and safety were performed according to GMP production. Table S2 offers a precise description of the validations and quality controls in the allogeneic cell production process for the ALOFEM study. Cells were packaged for shipment to the operating room with controlled temperature in a 7‐mL syringe at a dose of 20 million cells per mL (a total of 140 million cells).

#### 2.2.2. Surgical Intervention

The treatment involved implanting 140 million MSCs into the patient’s femoral head through forage controlled by fluoroscopy, as described by Gómez‐Barrena et al. [14]. Shortly, to avoid any risk of bacteremia, perioperative antibiotic treatment was administered according to the hospital protocol. After anesthesia, the patient was placed in supine position on a fracture table with sufficient traction in the lower limbs. The surgical approach was minimally invasive lateral to the proximal femur. A guidewire was inserted from the lateral cortex of the subtrochanteric femur to the lesion on the femoral head under AP and axial fluoroscopic control. Subsequently, a tunnel (forage) was drilled along the guidewire towards the femoral head with a 4 mm cannulated drill. Cells were injected directly into the forage tunnel in a single administration. In no case was the joint cavity perforated. Closure was performed according to the usual protocol.

##### 2.2.2.1. Forage Location

Postoperative anteroposterior radiographs were examined to measure the anatomical angle and the forage angle, taking as reference the intersection point of the cervicodiaphyseal angle (caput–collum–diaphyseal angle, CCD), and to locate the forage tunnel in the weight bearing area (WBA) thirds (I = section between anatomical and B1 angles, II = section between B1 and B2 angles, and III = section between B2 and B3 angles). The drilling crossed the sclerotic rim in all cases. All forages were placed inside the WBA‐I, except for the case 103R that was placed inside WBA‐III and case 104R that was placed inside WBA‐II. Table S3 provides the localization of the surgical forage, including its positioning towards the WBA.

### 2.3. Main Outcomes

The efficacy FU was performed at 12 months and the safety FU until 24 months. Radiological (anteroposterior and lateral x‐ray views) was performed at preoperative, 3, 6, and 12 months and MRI (coronal and transversal view) was performed at preoperative, 3, and 12 months. As part of the routine orthopedic FU, clinical evaluation including pain assessment and physical exam was performed at preoperative, 6 weeks, 3, 6, 12, and 24 months.

#### 2.3.1. Safety

Serious adverse events (SAEs), grade ≥ 3 adverse events (AEs) and AEs of special interest (AESIs) were recorded during the clinical trial. Events were recorded from patient inclusion until the end of the study participation. For the safety assessment of the IMP, the pre‐defined AESIs were AEs related to the administration procedures (i.e., surgery), malignancies (theoretical risk), and infections (theoretical risk).

#### 2.3.2. Efficacy

The efficacy was defined as bone regeneration maintaining head sphericity and/or avoiding progression of femoral head collapse in femoral heads with symptomatic osteonecrosis treated through a standard of care procedure plus a percutaneous injection of allogenic stem cells, derived from donor BM and expanded under GMP conditions. Nonhealing was defined as imaging (XR or MRI) progression to a more severe stage and/or receiving THR. Femoral head collapse was evaluated at 3, 6, and 12 months, as defined by a loss of femoral head sphericity, or propagation of the fracture with further depression of the femoral head upper pole, compared to preoperative femoral head. Second, progression towards a more severe ON grading, compared with preoperative imaging, was evaluated at 3, 6, and 12 months.

### 2.4. Secondary Outcomes

#### 2.4.1. Volumetric Assessment

The osteonecrosis volume (ONV) in the femoral head at 3 and 12 months in MRI, compared with preoperative status, was assessed on sets of coronal MRI sections on Digital Imaging and Communications in Medicine (DICOM) format using the OsiriX MD licensed software (Pixmeo, Switzerland) for MacOS (Apple, USA), following a standardized protocol. Regions of interest (ROIs) were outlined on each MRI slice manually, for the ON lesion and for the femoral head, following the anatomical contour. OsiriX automatically calculated the volume by multiplying the surface for each slice thickness and then summing up individual slice volumes. The final ONV was estimated as a percentage of the femoral head volume (FHV) following the formula : 100 × (ONV/FHV) [10].

#### 2.4.2. Lateral Spread of the ON Lesion

The coronal location of the necrotic lesion was evaluated for lateral progression, using the Japanese Investigation Committee classification system (JIC‐2001), on T1‐MRI or AP X‐ray, when MRI was unavailable. The lesion was preoperatively and postoperatively classified by the occupancy of the WBA, based on the central coronal section of the femoral head. Type A was allocated when the ON occupied the first, medial third of the WBA; type B when the second third of the WBA was occupied; type C1 when the ON reached the lateral third of the WBA but not surpassing the acetabular rim; and type C2, like type C1 but with the ON laterally surpassing the acetabular rim [15].

### 2.5. Statistical Analysis

All images were processed, measured, and classified with OsiriX software (Pixmeo, Switzerland) [16]. For statistical analysis, we used Stata Statistical Software: Release 12 (StataCorp, USA). In this descriptive study, results were shown under intention to treat data (ITT). The mean, standard deviation (SD), median, interquartile range (IQR), and the proportions were reported as appropriate. The statistical significance for comparison was defined with 95% of confidence. Fisher’s exact test was used for proportion comparisons. Mann–Whitney tests was used for ONV comparison between FU visits. The coronal location variables (as per JIC 2001) were described, and Fisher’s exact test differences were estimated, by FU visits.

## 3. Results

### 3.1. Safety Endpoint

No tumorous condition or cell‐related overgrowth was detected in any of the four patients after cell implantation.

Among the two patients treated under the clinical trial protocol scheme, a total of 10 SAEs or grade ≥ 3 AEs were reported and both patients recovered without sequelae. Regarding AESI, case 101 experienced two infection SAEs, both unrelated to the study treatment. A detailed description of serious or grade ≥ 3 AEs reported in the ALOFEM study can be found in the (Table S4).

For the compassionate treated cases, no postoperative complications related to the skin or scar, infections in the area, vascular complications or neurological complications were reported. The FU of case 103 was lost at 18 months due to death from relapse of her hematological disease. The relapse of the hematological malignancy was unrelated with the IMP.

### 3.2. Efficacy Endpoint and FU

Regeneration and progression of the ONFH were evaluated after a 1‐year FU. Clinical and radiological regeneration, maintaining head sphericity, was observed in 42% (3/7) of the treated hips. ITT data in Table 2 shows that 4/7 hips progressed to the next ARCO stage, one at 3 months, two at 6 months, and another one at 12 months. Of these progressed hips, two received THR at 10 and 13 months after surgical intervention, respectively. Late FU until Q4 2024, confirms that all four remaining hips have not received nor were expecting THR. The detailed clinical assessment, including pain and weight bearing, has been included in the Table S5.

**Table 2:** Radiological progression of the treated hips.

Hip code	ARCO stage				Radiological progression at 12mFU	THR at 12mFU	Latest FU
	PreOp	3mFU	6mFU	12mFU			
101L	IIIA	IIIA	IIIC	IIIC	Yes	No	13 mo (THR)
101R	IIB	IIC	IIC	IIC	Yes	No	4 yr (no THR, stage IIIA)
102L^a^	IIIB	IIIB	IIIB	IIIB	No	No	4 yr (no THR, stage IIIB)
102R	IIIA	IIB	IIIA	IIIA	No	No	4 yr (no THR, stage IIIA)
103R	IIIA	IIIA	nda	IIIB	Yes	No	18 mo (no THR, stage IIIB)^c^
104L	IIIA	IIIA	IIIB	n/a	Yes	Yes	10 mo (THR)
104R	IIB	IIB	IIB	IIB	No	No	5 yr (no THR)^b^

*Note*: Time is expressed in months (mo) or years (yr).

Abbreviations: FU, follow‐up; n/a, not applicable; nda, no data available; PreOp, preoperative; THR, total hip replacement.

^a^Protocol deviation: Steinberg stage IV.

^b^Image tests not available.

^c^Exitus at 18 months.

### 3.3. Secondary Endpoints

#### 3.3.1. Volumetric Assessment

As a percentage of the femoral head, the mean ± SD preoperative ONV was 40% ± 19% (median 47%, IQR = 29%), decreasing to a mean ± SD 28% ± 16 (median = 25%, IRQ = 18%) at 3 months FU but not significantly (Mann–Whitney test: *p* = 0.337). After 1 year of FU, the mean ± SD ONV was estimated at 32% ± 5% (median = 33%, IQR = 32%). Although 8% smaller than preoperatively, this was not statistically significant (Mann–Whitney test: *p* = 0.515), as illustrated in Figure 1a. The individual evolution of the ONV during FU is observed in Figure 1b.

Figure 1Osteonecrosis volume (ONV) as percentage of the femoral head, by follow‐up. (a) The boxplot illustrates the median ONV during follow‐up. Preoperatively, the median was estimated at 47% (IQR = 29%), 25% (IQR = 18%) at 3 months, and 33% (IQR = 32%) at 12 months. Although a reduction in ONV is observed, there is no statistically significant difference with preoperative volume at any FU time in the current, limited series (Mann–Whitney tests: 3 months *p* = 0.337; 12 months *p* = 0.515). (b) The line figure shows the evolution of the ONV per participant hip. The triangle symbols represent hips without ON progression to the next stage, while the circle symbols represent hips progressing or receiving THR (in red: 101 L, 104 L). All individual cases tended to reduce the volume of ON at 3 months FU, except for the hip 101R. One year after cell implantation, the trend was to increase the ON volume. MRI images of hips in case 104 were not available after 3 months follow‐up.

**Figure:**
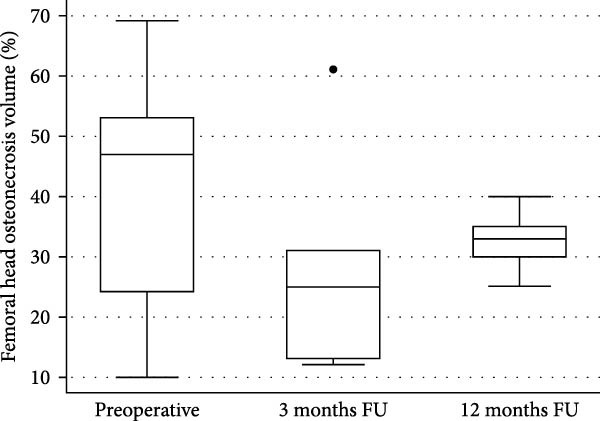
(a)

**Figure:**
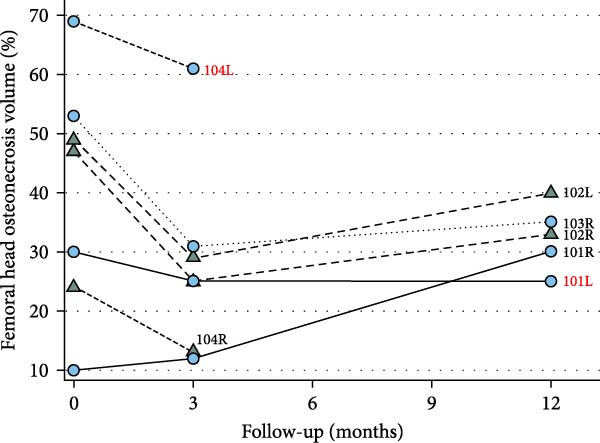
(b)

#### 3.3.2. Lateral Spread of the ONFH Lesion

**Figure 2:**
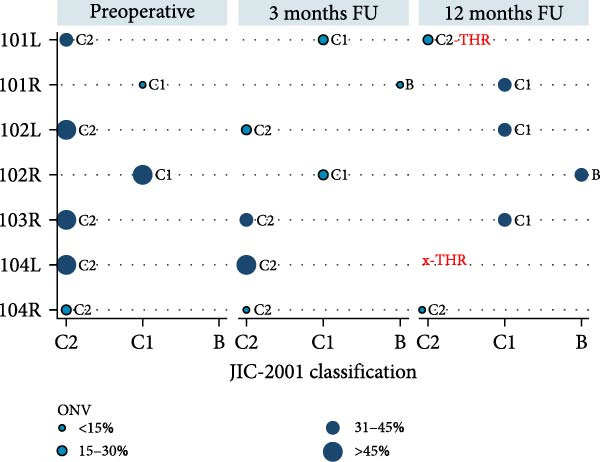
Evolution of JIC‐2001 classification and ONV% of each treated hips, by follow‐up time. JIC‐2001 classification: type A when ON occupies the first third of the weight bearing area (WBA); type B when ON occupies the second third of the WBA; type C1 when ON occupies the final third of the WBA but not surpassing it; and type C2, which is like type C1 but laterally surpasses the WBA and the acetabular rim. Data were obtained from MRI images for all the cases, except for hip 104R at 12 months FU in which x‐rays were used for JIC‐2001 classification, but ONV data were unavailable, then using the same as at 3 months FU.

The evolution of coronal plane distribution (as per the JIC‐2001 classification) is presented in Figure 2 for the participant cases during FU time. Preoperatively, 5/7 (71%) of the ON lesions were classified as C2, and 2/7 (29%) were C1. Three months after cell implantation, 4/7 (57%) of ON lesions remained as C2, 2/7 (29%) were classified as C1, and 1/7 (14%) were classified as B. After a 1‐year FU, 2/6 (33%) of the ON lesions were C2, 3/6 (60%) were C1, and 1/6 (20%) turned to B. No proportion differences were observed from preoperative at any FU time (Fisher’s exact test: 3 months *p* = 0.592; 12 months *p* = 0.755).

Finally, Figure 3 illustrates the radiological evolution of hips 103R and 101 L, while Figure 4 describes the pathology of the explanted hip 101 L.

Figure 3Radiological evolution of hips 101L and 103R. From left to right: Preoperative, 3 months FU, and 12 months FU. (a) Hip 103R starts with an ONV of 53%, as a percentage of the femoral head, being classified as C2 by the JIC‐2001 system. The ONV was reduced to 30% after three months since surgery, but the classification remains C2. After one year of follow‐up, the ONV was 35% but classified as C1. The yellow arrow shows the bone formation observed in zone III of the weight bearing area (WBA). Long‐term follow‐up confirms that THR was not received after 18 months. (b) Hip 101L starts with an ONV of 30%, as a percentage of the femoral head, being classified as C2 at preoperative. The classification does not change during follow‐up. After one year of the surgical intervention, a reduction of 5% of the ONV is observed, but not enough to complete bone regeneration in zone III of the WBA. Finally, the femoral head collapsed, and at 13 months the patient received a THR. The explanted head is described in Figure 4.

**Figure:**
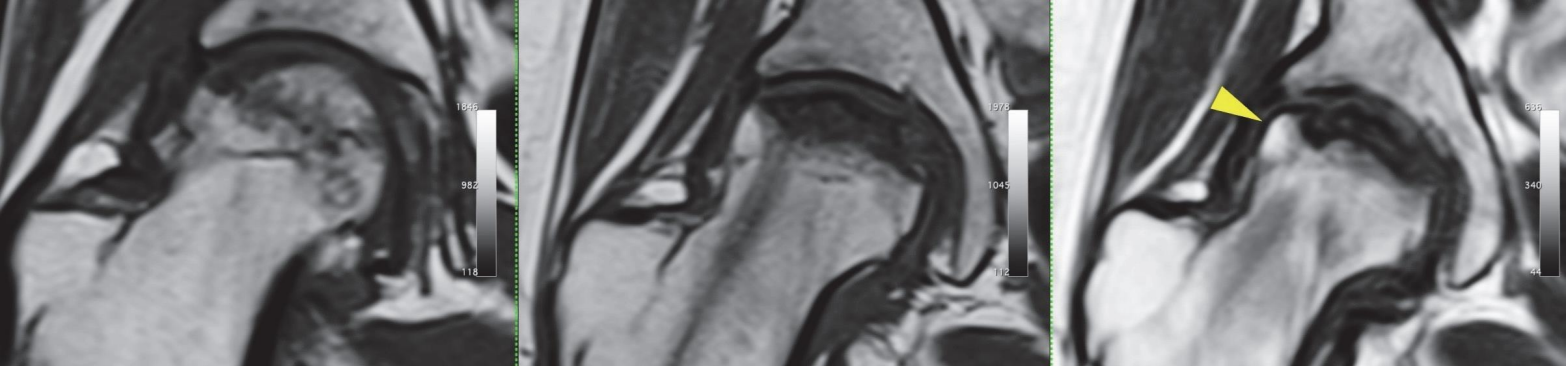
(a)

**Figure:**
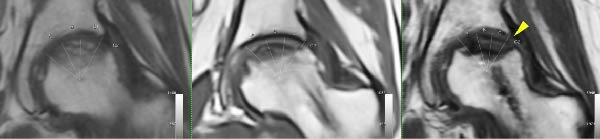
(b)

Figure 4Pathology report of the retrieved femoral head in case 101L. (a) The femoral head measured 4.5 cm × 4.4 cm × 3.5 cm. It had a spherical morphology and a smooth surface with intact articular cartilage, although a depressible area can be seen in the weight bearing area. In the coronal section, made with the EXAKT mechanical saw at 1000 m/min with light irrigation, the depressible area corresponded to a subchondral area of yellowish color and less consistency, circumscribed in depth by bone tissue arranged in a whitish band. The sections were fixed in 10% neutral buffered formalin for 48 h, then washed under running water and decalcified with 7% nitric acid for 10 h. They were embedded in paraffin after dehydration in alcohols at increasing concentrations. Histological sections were made with a thickness of 4 microns and stained with hematoxylin and eosin. Histological analysis shows (b) Intact articular cartilage with preserved architecture, equidistant and orderly arrangement of chondrocytes in its matrix, and a subtle tendency towards eosinophilia of the chondral matrix in its deep third (OSN × 20). (c) The subchondral bone is necrotic, with empty osteocyte lacunae and no adjacent viable intertrabecular tissue (OSN × 100). (d) A breach in continuity at the subchondral spongy tissue running parallel to the curvature of the articular surface is observed (OSN × 100). (e) Beneath the area of bone necrosis, fibrochondral tissue is arranged in a band with evident endochondral ossification towards the epiphyseal epicenter (OSN × 100). (f) In this area, abundant vascularization and marked osteoblastic activity are observed (OSN × 200).

**Figure:**
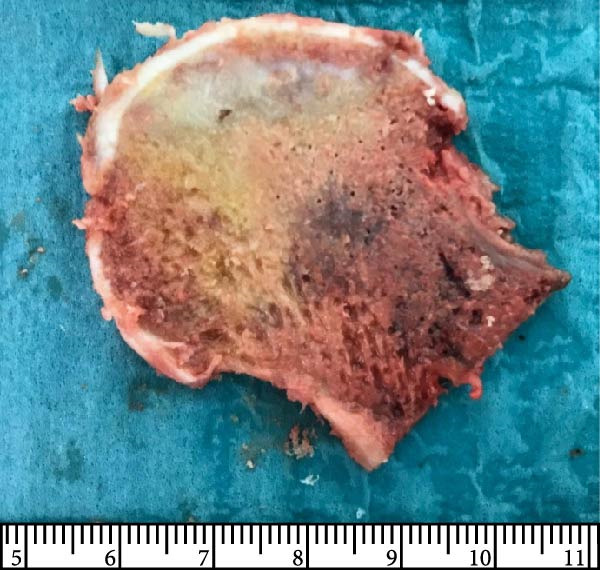
(a)

**Figure:**
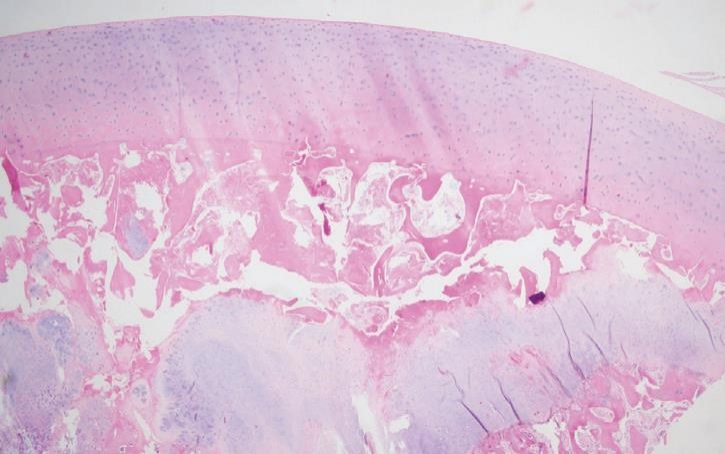
(b)

**Figure:**
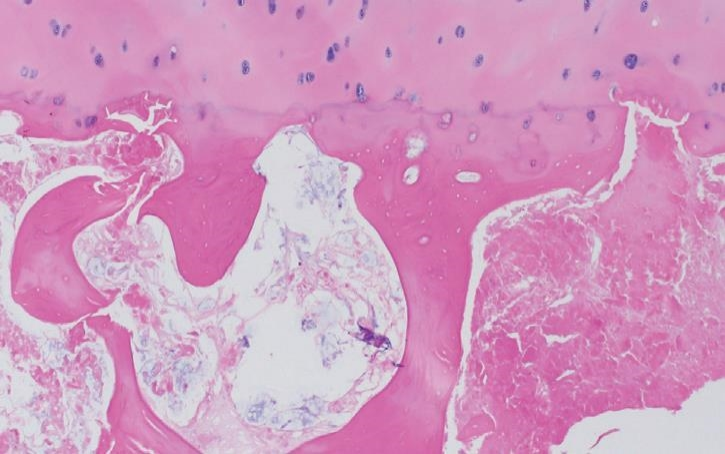
(c)

**Figure:**
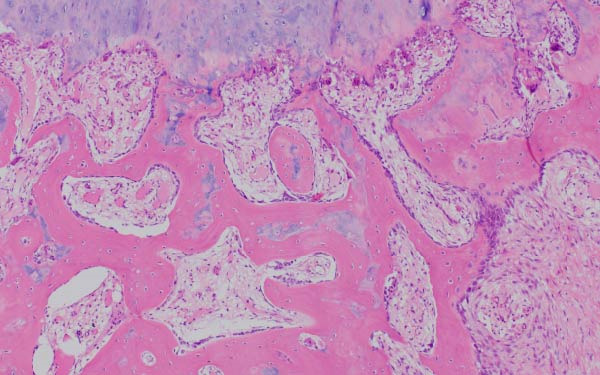
(d)

**Figure:**
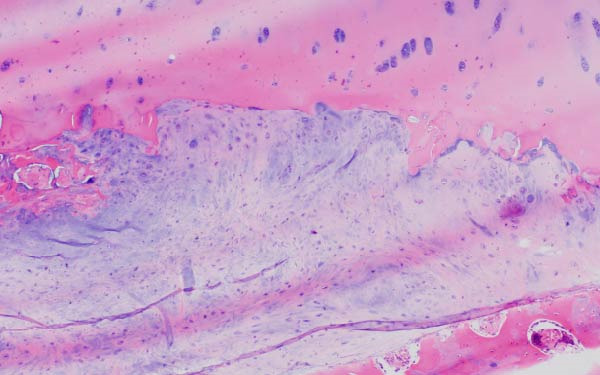
(e)

**Figure:**
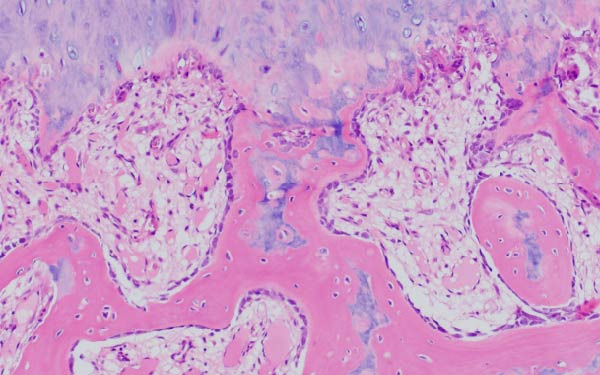
(f)

## 4. Discussion

This pilot clinical study includes four young, female patients with a history of malignant disease who developed bilateral ONFH secondary to corticosteroid therapy, in addition to other concomitant clinically severe conditions. The seven hips included in the study were in advanced stages of progression, and the patients were proposed to undergo a THR procedure as an option at the time of recruitment. The promising cell therapy from autologous donation of BM was not possible due to their malignancy history; therefore, there were no alternative treatment options for these young patients except experimental methods (in this case, implantation of allogeneic MSC) to preserve the femoral head, alleviate symptoms, maintain mobility, and postpone the replacement surgery. For safety reasons, the received allogenic treatment was manufactured from the BM of compatible donors and preselected from previous treatment needs.

The fact that we are presenting a phase I clinical trial, first‐in‐human under these specific conditions and for this underrepresented patient’s group is the justification of this small sample size. While the efficacy is just suggested, the main aim of the phase I clinical trial is not to prove efficacy but rather to confirm safety and observe at least some desired bone regeneration after a minimally invasive approach in those advanced cases without other potential treatment.

No AEs that make it necessary to re‐evaluate the benefit/risk balance of the IMP in this indication were reported. Specifically, no tumorous condition or cell‐related overgrowth was detected in any of the four patients after cell implantation. Long‐term FU (upto 4 years) did not detect any safety concerns regarding the allogeneic treatment. These results align with those reported with the autologous treatments for the same condition [9, 16].

Available evidence strongly supports the clinical safety of allogeneic MSC not only in immunosuppressed conditions [17] but also in immunocompetent subjects treated for bone and cartilage diseases. However, none of the participants in this study were under immunosuppressive treatment at the time of the trial. The local injection of allogeneic MSCs has proven safe and effective for the treatment of chronic knee osteoarthritis in a phase II clinical study by Gupta and colleagues (2016) [18], the most relevant side effects being mild local pain and swelling. Another randomized controlled multicentre phase I–II trial with allogeneic BM‐derived MSC in the same clinical context demonstrated, by means of quantitative magnetic resonance imaging, the healing of partial articular cartilage and no major AEs. Reassuring safety results were also reported using allogeneic adipose tissue (AT)‐derived MSC [19] and umbilical cord (UC)‐derived MSC [20].

Bone regeneration in the femoral head is observed after treatment with allogeneic expanded MSC from BM at a dose of 140 × 10^6^ MSC, injected into the femoral head through a minimally invasive approach after forage. In general terms, the IMP induced initial bone formation, and the ONV was reduced 3 months after surgical intervention. However, after 1 year, the ONV increased again. This finding supports the proposal of Gomez‐Barrena et al. [10] to include a second cell dose when the residual ONV after 3 months of FU is higher than 15% of the femoral head. In our series, only two out of seven hips achieved this threshold at 3 months, which leads us to hypothesize that a second dose at 3 months could have been beneficial to effectively reduce the ONV in advanced disease. Further studies are needed to evaluate this repeated injection as the best strategy to improve bone regeneration results.

We applied the technique to patients suffering from hematological disorders because they frequently sustain bone osteonecrosis in advanced stages due to intensive corticotherapy, and because the BM reaction may be affected after hematopoietic stem cell transplant. We agree that the mechanism behind the benefits of mesenchymal stem cell therapy in hematological diseases treated by HSCT remains unclear and could be a future direction of research.

Comparing the autologous and allogeneic cell treatment, the observed bone regeneration with allogeneic cells in our study was similar to that with autologous cells, especially in advanced stages with high volumes of osteonecrosis [21, 22]. In our series, only one hip was included with ONV under 15% and was not expected to convert to THR. For the remaining six hips, two required THR after 1 year. The rate of conversion is then 33%, which is the rate expected for femoral heads with ONV over 15%, as described by Kuroda et al. [23]. The overall rate of conversion in our study is 28% (2/7) at 1 year and 33% (2/6) after 4 years. In terms of efficacy, our study shows similar results as other in the range of 70%–90% of cell therapy efficacy to avoid the collapse or conversion to THR [24–26]. Regarding allogenic treatment, Hernigou et al. [27] did not find differences in bone regeneration, comparing BM aspirate concentrate (BMAC), allogenic (Allo CT), and autologous expanded cells (Auto Exp CT) in early osteonecrosis (stage ARCO I) of young patients (less than 30 years). They did not find significant differences in treatment outcomes among treatments with hip repair rates of 72%, 75%, and 73%, respectively. The reductions in volume of hip osteonecrosis were also similar; specifically, for BMAC, the preoperative ONV decreased from a mean of 28%–9.2%, while the Allo CT cohort saw a decline from 25.3% to 9.3% (paired *t*‐test; *p* = 0.32) [27]. These findings are similar to those observed in our study, with regeneration of approximately 70% of the osteonecrosis lesion, although with higher volumes of osteonecrosis and more severe stages. Validation of these findings through larger randomized controlled trials is necessary before a strong conclusion on efficacy can be established.

The location of the ON in the femoral head was also assessed in our study. According to Sugano et al. [15], the regeneration of the WBA, particularly the lateral third, could avoid collapse or prevent the conversion to THR. Results from our study are consistent with this assumption, observing that 2/7 of the femoral heads finally collapsing and receiving THR did not generate bone in the lateral third of the WBA. One more hip also classified as C2 preoperatively (beyond the acetabular rim) still avoided collapse after 1 year, although evaluated just with X‐rays, which could induce error. Four heads reduced their osteonecrosis coronal extension and were classified as C1 (within the acetabular rim). Although one hip progressed to the next stage, none collapsed as no lateral spread was observed.

Finally, the pathology results in one hip undergoing THR 1 year after cell therapy treatment proved changes occurring in the bone tissue, developing cartilage at the place of osteonecrosis and far lateral in the head. This suggests the presence of regeneration with insufficient ossification and the presence of a nonhealed fracture in the lateral region of the head, associated with femoral head collapse. Healing of the ON may only be obtained if bone is regenerated laterally in the head to avoid collapse, which is consistent with placing the forage tunnel during surgery in the most lateral region of the femoral head.

### 4.1. Limitations

The main limitation of this pilot study is the small sample size. Although it has shown different trends in safety and efficacy, and a rationale to treat severely affected young patients lacking an alternative therapy, more cases are required to confirm the findings and obtain significance. The second limitation is that the results are inconclusive due to the lack of a comparative group; therefore, we cannot ignore the potential confounding factors that could affect the reduction of the ONV. Although this phase I clinical trial provides data on feasibility, safety, and potential efficacy, a larger, randomized clinical trial would be required to support the spread of this treatment. A third limitation is the limited precision in the ONV estimation. ROIs were manually defined by one single experienced researcher, but accuracy and repeatability were not evaluated. Even if the influence of this limitation is probably scarce in the detailed case analysis, it may affect the lack of significance. A fourth limitation is due to the complex concomitant treatments in the patients, and the necessity of reintroducing corticosteroids due to the patient’s severe disease or relapse, interfering with the outcome, as reported in noninterventional studies [28–30]. Finally, as a fifth limitation, results may be influenced by the bilaterality condition, which could also affect the time for regeneration, as described by Koruda et al. [23].

## 5. Conclusions

The allogeneic implantation of expanded MSC in young patients to prevent conversion to a THR or collapse of the femoral head due to severe osteonecrosis is feasible, and no safety concern was observed neither at 1‐year FU nor in the longer‐term FU upto 4 years. The results suggested, but were unable to prove, early efficacy at this stage of the treatment development.

## Disclosure

The funders had no role in the design of the study; in the collection, analyses, or interpretation of data; in the writing of the manuscript; or in the decision to publish the results.

## Conflicts of Interest

The authors declare no conflicts of interest.

## Funding

This study was funded by the Spanish Ministry of Science and Innovation and Instituto de Salud Carlos III (ISCIII) through the State Plan for Scientific and Technical Research and Innovation, Strategic Actions in Health (AES), Project Number PI17/01844.

## Supporting Information

Additional supporting information can be found online in the Supporting Information section.

## Supporting information


**Supporting Information** Figure S1. CONSORT flow diagram illustrating patient screening, enrollment, follow‐up, and assessment in the ALOFEM clinical study. Table S1. Inclusion and exclusion criteria used for the selection of candidate patients in the ALOFEM clinical study. Table S2. Description of the allogeneic cell manufacturing process applied in the ALOFEM clinical study, including validation and quality control procedures. Table S3. Anatomical localization of the hip forage within the weight bearing area (WBA). Table S4. Summary of serious adverse events (SAEs) or adverse events of grade ≥3 reported during the ALOFEM clinical study, corresponding to cases 101 and 102. Table S5. Clinical outcome assessment related to pain and weight‐bearing capacity throughout the ALOFEM clinical study follow‐up.

## Data Availability

The data that support the findings of this study are available on reasonable request from the corresponding author. The data are not publicly available due to privacy or ethical restrictions.
