# Supplementary material for: Safety, Feasibility, and Preliminary Efficacy of Allogeneic MSCs to Treat Advanced Femoral Head Osteonecrosis (ALOFEM): A Pilot Study in Young Onco‐Hematological Patients
Source: Stem Cells Int. 2026 Jan 5;2026:1986839. doi: 10.1155/sci/1986839 (PMC12771626; doi:10.1155/sci/1986839)
Supplement: Supplementary file 1 — Supporting Information Figure S1. CONSORT flow diagram illustrating patient screening, enrollment, follow‐up, and assessment in the ALOFEM clinical study. Table S1. Inclusion and exclusion criteria used for the selection of candidate patients in the ALOFEM clinical study. Table S2. Description of the allogeneic cell manufacturing process applied in the ALOFEM clinical study, including validation and quality control procedures. Table S3. Anatomical localization of the hip forage within the weight bearing area (WBA). Table S4. Summary of serious adverse events (SAEs) or adverse events of grade ≥3 reported during the ALOFEM clinical study, corresponding to cases 101 and 102. Table S5. Clinical outcome assessment related to pain and weight‐bearing capacity throughout the ALOFEM clinical study follow‐up. [file SCI-2026-1986839-s001.docx]

Supplementary Materials

**Figure S1. CONSORT diagram of the ALOFEM study**

## Follow-Up and Assessment

## Treated

Screened (n=10)

- Prior to eligibility assessment (n=7)

- Compassionate treated cases (n=3)

Excluded (n=3)

- Relapse of haematological disease (n=2)

- Preoperative corticoid treatment (n=1)

## Screened

Protocol deviation (n=1)

- Selection criteria (Steinberg stage IV-ARCO IIIC)

Included and surgically treated with

140x10^6^ BM-derived allogeneic MSC

(n=7)

**Safety:**

- One-year safety (n=7)
- Long term (until 4y) safety (n=4)

**Efficacy:**

- One-year efficacy PP (n=6)
- One-year efficacy ITT (n=7)
- Long term (until 4y) efficacy (n=4)

Lost 2y follow-up:

- THR (n=2)

- Unrelated exitus (n=1)

*BM: Bone Marrow; MSC: Mesenchymal Stem Cells; THR: Total Hip Replacement; PP: Per Protocol; ITT: Intention To Treat*

**Table S1.** **ALOFEM clinical trial selection criteria of candidate patients**

| **No.** | **Description** |
| --- | --- |
| ***Inclusion criteria*** | |
| 1. Age 8 to 55 2. Both sexes 3. Symptomatic ONFH (Ficat and Arlet 0, 1, 2 or 3 or ARCO stages 0, I, IIA, IIB, IIC, IIIA, IIIB, IIIC or Steinberg stages 0, I, II, III.) 4. Having received HSCT due to hematologic disease (in remission) 5. Being able to provide it and able to understand and accept the study constraints and sign the informed consent 6. Medical coverage under the Spanish National Health System 7. Patients that, alternatively, would have received forage | |
| ***Exclusion criteria*** | |
| 1. Disease in relapse 2. Active infection of any location and aetiology 3. Surgical contraindication of any cause 4. Pregnancy 5. Breastfeeding women and women who are of childbearing age and not practicing adequate birth control 6. Stage 4 (Ficat and Arlet) or IV and above (Steinberg) or ARCO IV, with severe femoral head osteonecrosis associated to acetabular changes, primarily based on diagnosis by imaging (X-rays, MRI) 7. Flattening or collapse of the femoral head (Steinberg stage IV) or articular cartilage collapse at the time of core decompression surgery 8. Any tumour infiltration with short-term life risk; stress or pathological fracture of the femoral neck 9. Tumour infiltration with short-term life risk 10. Stress or pathological fracture of the femoral neck 11. Traumatic osteonecrosis 12. Non-osteonecrosis metabolic bone diseases (particularly Paget's disease of bone, osteogenesis imperfecta, primary hyperparathyroidism, monostotic or polyostotic fibrous dysplasia, McCune-Albright syndrome and osteopetrosis) 13. Any active bisphosphonate treatment (or 3 months before the trial, except in case they are used in the treatment of an acute disorder that requires prompt management, e.g., severe hypercalcemia, severe bone pain) 14. Any evidence of HIV, Hepatitis B or Hepatitis C infection (confirmed by serology or PCR) 15. Known allergies to products involved in the production process of MSC 16. History of regular alcohol consumption exceeding 2 drinks/day (1 drink = 150 mL of wine or 360 mL of beer or 45 mL of hard liquor) within 6 months of screening and/or history of illicit drug use within 6 months of screening and/or history of illicit drug use 17. MRI incompatible internal devices (pacemakers, aneurysm clips, etc.) | |
| *ONFH=Osteonecrosis of the Femoral Head; HSCT=Hematologic Stem Cell Transplantation; MRI=Magnetic Resonance Imaging; MSC=Mesenchymal Stem Cells* | |

**Table S2. Allogeneic cell production process for the ALOFEM study: Validations and Quality Controls**

| Validation and Quality Control variables | Hip code (donor*) | | | |
| --- | --- | --- | --- | --- |
|  | case 101  (parent) | case 102 (parent) | case 103  (parent) | case 104  (sibling) |
| **Characteristics of the donor bone marrow aspirates** | | | | |
| Date of harvest | 02/03/2020 | 04/11/2020 | 26/04/2017 | 22/10/2019 |
| Initial BM volume (ml) | 85 | 77 | 65 | 50 |
| Cell concentration (10^6^/ml) | 22.50 WBC | 10.58 WBC | 15.10 WBC | 16.90 WBC |
| Total No. of cells (x10^6^) | 1912.50 WBC | 814.66 WBC | 981.50 WBC | 845.00 WBC |
| Cell viability (%) | 95.6 | 95.3 | 93 | 96 |
| CFU-F frequency (x10^6^ BM WBC) | 19 | 58 | 84 | 114 |
| Bacterial analysis | N | N | N | N |
| Mycoplasma | N | N | N | N |
| **Doubling time and the number of population doublings in P0 and P1** | | | | |
| Doubling time in P0 [h] | 19.96 | 21.41 | 23.07 | 14.47 |
| Doubling time in P1 [h] | 28.84 | 28.58 | 22.49 | 37.99 |
| Number of population doublings in P0 | 16.83 | 15.69 | 14.56 | 14.92 |
| Number of population doublings in P1 | 5.83 | 6.72 | 6.4 | 6.32 |
| Cumulative population doublings | 22.66 | 22.41 | 20.96 | 21.24 |
| **Yield (calculated as harvested MSC/μl seeded BM on day 0 of expansion) and overall harvest of expanded MSC** | | | | |
| MSC/µl BM aspirate in P0 | 12489 | 8125 | 7660 | 14970 |
| MSC/µl BM aspirate in P1 | 25509 | 22292 | 25534 | 26946 |
| Overall harvest (Millions of MSC) | 288 | 535 | 430 | 405 |
| **Surface markers to proof identity and purity of expanded cells of Passage 0** | | | | |
| % CD34 positive cells | 0.12 | 0.11 | 0.1 | 0.09 |
| % CD45 positive cells | 0.5 | 0.25 | 0.17 | 0.52 |
| % CD73 positive cells | 99.53 | 98.92 | 99.34 | 98.13 |
| % CD90 positive cells | 99.61 | 99.83 | 99.41 | 99.64 |
| % CD105 positive cells | 97.97 | 99.07 | 97.57 | 96.69 |
| % MHC cII positive cells | 0.55 | 0.2 | 0.1 | 0.32 |
| **Surface markers to proof identity and purity of expanded cells of Passage 1** | | | | |
| % CD34 positive cells | 0.1 | 0.08 | 0.04 | 0.09 |
| % CD45 positive cells | 0.13 | 0.11 | 0.17 | 0.16 |
| % CD73 positive cells | 99.31 | 99.62 | 98.76 | 99.76 |
| % CD90 positive cells | 99.89 | 99.99 | 99.85 | 99.99 |
| % CD105 positive cells | 97.74 | 98.62 | 97.6 | 99.01 |
| % MHC CII positive cells | 0.68 | 0.09 | 0.18 | 0.13 |
| **Viability of cells in starting material (bone marrow aspirate), P0 and P1 MSC** | | | | |
| % Viable cells in aspirate | 95.6 | 95.3 | 93 | 96 |
| % Viable cells after harvest of P0 | 100 | 95.3 | 99 | 96.4 |
| % Viable cells after harvest of P1 | 99.5 | 99.1 | 97.2 | 99.4 |
| **Quality controls and release parameters on microbiological safety** | | | | |
| Microbial testing:  -BM  -P0  -P1 | N  N  N | N  N  N | N  N  N | N  N  N |
| Mycoplasma testing P1 | N | N | N | N |
| Endotoxin testing P1 | N | N | N | P^1^ |
| *BM: Bone Marrow; WBC: leukocyte count; CFU-F: colony-forming units-fibroblast; N: Negative; P: Positive;*  *MSC: Mesenchymal Stem Cells; *Different sibling that HLA donation; ^1^* *Not a batch release criterion* | | | | |

**Table S3. Localization of the forage inside the Weight Bearing Area (WBA)**

| Hip  code | Anatomical angle | B1  angle | B2  angle | B3  angle | Forage  angle | Forage localization in the WBA |
| --- | --- | --- | --- | --- | --- | --- |
| 101L | 138.1 | 145.6 | 153.1 | 160.6 | 141.3 | I |
| 101R | 131.6 | 138.2 | 144.9 | 151.6 | 134.5 | I |
| 102L | 141.5 | 147.9 | 154.3 | 160.7 | 142.3 | I |
| 102R | 135.7 | 144.0 | 152.3 | 160.6 | 137.3 | I |
| 103R | 138.9 | 144.4 | 149.9 | 155.5 | 150.4 | III |
| 104L | 135.0 | 141.5 | 148.1 | 154.6 | 136.3 | I |
| 104R | 137.1 | 142.6 | 148.1 | 153.7 | 144.6 | II |

**Table S4. Serious or grade ≥3 Adverse Events reported in the ALOFEM study: Cases 101 and 102**

| **System Organ Class*** | **Preferred term*** | **n** |
| --- | --- | --- |
| General disorders and administration site conditions | Pyrexia | 3 |
| Skin and subcutaneous tissue disorders | Pruritus | 1 |
| Gastrointestinal disorders | Esophageal ulcer | 1 |
| Infections and infestations | Catheter site infection | 1 |
|  | Clostridium difficile infection | 1 |
| Reproductive system and breast disorders | Genial labia adhesions | 1 |
| Surgical and Medical procedures | Hip surgery | 1 |
| Renal and urinary disorders | Hydronephrosis | 1 |
| **MedDRA version 27.1* | | |

**Table S5. Clinical assessment: Pain and weight bearing**

|  |  | Hip code | | | | | | |
| --- | --- | --- | --- | --- | --- | --- | --- | --- |
|  | Visit | 101L | 101R | 102L | 102R | 103R | 104L | 104R |
| ***Pain at rest ^(1)^*** | Preoperative | 5 | 0 | 0 | 0 | 4 | 4 | 2 |
|  | 3 months FU | 10 | 4 | 0 | 0 | 2 | *nda* | *nda* |
|  | 6 months FU | 10 | 2 | 0 | 0 | 2 | 4 | 3 |
|  | 12 months FU | 9 | 3 | 0 | 0 | 2 | 8 | 3 |
|  | 24 months FU | *n/a* | 3 | 0 | 0 | *n/a* | *n/a* | *nda* |
| ***Pain under weight-bearing ^(1)^*** | Preoperative | 8 | 4 | 3 | 3 | 4 | 5 | 4 |
|  | 3 months FU | (*) | (*) | 0 | 0 | 5 | *nda* | *nda* |
|  | 6 months FU | (*) | (*) | 0 | 0 | 4 | 8 | 6 |
|  | 12 months FU | (*) | (*) | 0 | 0 | 2 | 10 | 3 |
|  | 24 months FU | *n/a* | 8 | 0 | 0 | *n/a* | *n/a* | *nda* |
| ***Monopodal weight-bearing ^(2)^*** | Preoperative | No | No | Yes | Yes | No | Yes | Yes |
|  | 3 months FU | No | No | Yes | Yes | No | Yes | Yes |
|  | 6 months FU | No | No | Yes | Yes | Yes | Yes | Yes |
|  | 12 months FU | No | No | Yes | Yes | Yes | Yes | Yes |
|  | 24 months FU | *n/a* | Yes | Yes | Yes | *n/a* | *n/a* | *nda* |
| *^(1)^ Likert scale from 0 to 10, where zero means no pain at all and ten means the worst pain ever felt. Pain is considered with values over or equal to 4 points.*  *^(2)^ Weight-bearing with monopodal stance without crutches*  *(*) The patient did not walk; therefore, it was not possible to evaluate pain under weight-bearing*  *nda: No data available; n/a: Not applicable* | | | | | | | | |
